# Sleep quality and general health status of employees exposed to extremely low frequency magnetic fields in a petrochemical complex

**DOI:** 10.1186/2052-336X-12-78

**Published:** 2014-04-29

**Authors:** Mohammad Reza Monazzam, Monireh Hosseini, Laleh Farhang Matin, Habib Allah Aghaei, Hossein Khosroabadi, Ahmad Hesami

**Affiliations:** 1 Department of Occupational Hygiene, School of Public Health and Center for Air Pollution Research (CAPR), Institute for Environmental Research (IER), Tehran University of Medical Sciences, Tehran, Iran; 2 Department of Physics, Faculty of Basic Sciences, Islamic Azad University- Tehran North Branch and Center for Air Pollution Research (CAPR), Institute for Environmental Research (IER), Tehran University of Medical Sciences, Tehran, Iran; 3 Department of Physics, Faculty of basic sciences, Islamic Azad University- Tehran north branch, Tehran, Iran; 4 Department of Occupational Health, School of Public Health, Tehran University of Medical Sciences, Tehran, Iran; 5 National Petrochemical Company, Tehran, Iran

**Keywords:** Electromagnetic fields, ELF, Quality of sleep, General health

## Abstract

**Background:**

Advances in science and technology of electrical equipment, despite increasing human welfare in everyday life, have increased the number of people exposed to Electro-Magnetic Fields (EMFs). Because of possible adverse effects on the health of exposed individuals, the EMFs have being the center of attention. This study was performed to determine possible correlation between Extremely Low Frequency Electro-Magnetic Fields (ELF EMFs) and sleep quality and public health of those working in substation units of a petrochemical complex in southern Iran.

**Materials and method:**

To begin with, magnetic flux density was measured at different parts of a Control Building and two substations in accordance with IEEE std 644–1994. Subsequently, the questionnaires “Pittsburgh Sleep Quality Index” (PSQI) and “General Health Quality (GHQ)” were used to investigate relationship between ELF exposure level and sleep quality and public health, respectively. Both questionnaires were placed at disposal of a total number of 40 workers at the complex. The filled out questionnaires were analyzed by *T*-test, Duncan and the Chi-square tests.

**Results:**

The obtained results revealed that 28% of those in case group suffered from poor health status and 61% were diagnosed with a sleep disorder.

However, all members in control group were in good health condition and only 4.5% of them had undesirable sleep quality.

**Conclusion:**

In spite of a significant difference between the case and control groups in terms of sleep quality and general health, no significant relationship was found between the exposure level and sleep quality and general health. It is worth noting that the measured EMF values were lower than the standard limits recommended by American Conference of Industrial Hygienists (ACGIH). However, given the uncertainties about the pathogenic effects caused by exposure to ELF EMFs, further epidemiological studies and periodic testing of personnel working in high voltage substations are of utmost importance.

## Introduction

Due to a substantial increase in the use of electrical energy in everyday life, number of people exposed to these fields in public or at workplace has increased dramatically [1-3].

Due to concerns about the possible harmful effects of exposure to EMFs, this physical agent has been the centre of attention in recent years [4,5]. Effects of EMFs on living organisms at different frequencies have long been under investigation by researchers worldwide. Kostoff and Lau in 2013 examined the scope of the accumulated effects on biological and health systems caused by simultaneous exposure to electromagnetic fields/radiation. They finally concluded although, there is a wide range of potential effects in which EMF plays a supportive or negative role; however, community consensus does not exist on these potential effects, either beneficial or adverse [6].

Another research by Kroupová et al. in 2007 studied the effects of LFM on cytoskeleton and on the structure of chromatin in human cells. They could finally find no influence on higher order chromatin structure, however, certain changes on the level of cytoskeleton was detected by them [7].

A survey research was also done by Cârstea in 2012 on the impact of EMFs of the large power cables on human health. They could finally present an algorithm to stimulate the adverse health impacts [8]. What approved by all is harmfulness of overexposure to the EMFs. Anyhow, further researches seem necessary to study the effects of different exposure levels. Many studies have shown correlation between occupational and non-occupational exposure levels to EMFs and the risk of some types of cancer including leukemia, brain tumors and breast cancer. Such a relationship has not been confirmed by all.

On the contrary, the findings of dozens of studies revealed non-cancer effects of exposure to magnetic fields. A study by Chung-Yi et al. in 1996 showed that exposure to high EMFs can cause sleep disorders in women [9]. Bianchi and Thomas in 2013 also studied the current clinical standard for enumerating sleep physiology by EMF, from which basic sleep–wake stages are determined. It was found that the complexity of sleep physiology has inspired alternative metrics that are providing additional perceptions into the rich dynamics of sleep [10]. In another research by Hashish et al. in 2008, it was investigated the effects of continuous whole-body exposure to EMF on liver and blood parameters in mice. Their findings revealed relation between the ELF-EMF exposure and the oxidative stress through distressing redox balance which leads to physical disturbances [11].

Savitz et al. (1999) found a significant relationship between exposure to ELF EMFs and death from arrhythmia and Acute Myocardial Infarction (AMI) while no significant relationship they could find between ELF EMFs and is chemic heart disease [12]. In a survey by Sahl et al. (2002), no correlation was observed between AMI or chronic coronary heart disease and cumulative exposure to ELF-MF [13]. Noonan in 2002 performed a case–control study to investigate possible relationship between neurodegenerative diseases and occupational exposure to magnetic fields. They could finally find evidences of correlation between occupational exposure to MFs and Parkinson’s disease. However, no relationship was reported by them on exposure to magnetic fields and Alzheimer’s disease [14]. In a cohort study by Qiu et al. (2004) done in Stockholm, the risk of Alzheimer’s disease and occupational exposure to magnetic fields was investigated. Their findings claimed that long-term occupational exposure to intense EMFs can increase the risk of dementia and Alzheimer’s diseases among men while similar exposure conditions did not show any increase in the risk of Alzheimer’s diseases in women [15]. Davanipour et al. (2007) found a positive correlation between exposure to EMF waves and Alzheimer’s debases [16]. Seidler et al. in 2007 through a case–control study indicated that there is no significant correlation between exposure to magnetic fields and dementia, vascular dementia and Alzheimer’s and diseases [17]. In a study by Yoausefi and Nasiri (2006) on employees of high voltage substations in Tehran, it was reported symptoms of depression, paranoia, obsessive-compulsive disorder, interpersonal sensitivity, anxiety, aggression, phobia or psychosis among employees [18]. Sharifi et al. (2009) showed that long-term exposure to magnetic fields, despite being below the limits recommended by the International Commission on Non-Ionizing Radiation Protection (ICNIRP), can cause exacerbation of intensify psychiatric disorders including sleep disorders [19].

Given the widespread and growing trend of releasing ELF EMFs and recent reports on their adverse effects on different body systems, this study was done to investigate possible correlation between ELF EMFs and the general health and sleep quality of employees working at two power substations in a petrochemical company in southern Iran.

## Material and methods

In this study, in order to assess exposure level of personnel to high voltage substations, magnetic flux density was measured using grid method at different parts of the Control Building and two substations located in a petrochemical complex. Number of measurement points was 126 at Ethylene Substation, 24 at Boiler Substation and 70 at Control Room. Changing number of measurement points at each location is due to differences in dimension and shape of the rooms as well as layout of equipment and power panels. All measurements were done in duplicate at each grid point according to IEEE STD 644–1994 in normal site conditions [20]. The measurement instrument was a uniaxial HI-3604 ELF Survey Meter manufactured by Holaday Company. The device was held by the operator at the height of 1 m from the ground towards three axes X, Y and Z. The maximum value measured in three directions (B_max_) was considered as the magnetic field strength at each point.

The device has the capability of measuring both 50/60Hz- electric and magnetic fields from power transmission and distribution lines and equipment. It has a sensitivity of about 1 V/m – 199 KV/m against electric fields and 0.1 mG – 20 G against magnetic fields [21]. The values of the magnetic flux density were compared with limits recommended by ACGIH [22]. Reviewing information contained in career records, the type of jobs and the numbers of people employed in each unit were specified. Table 1 presents records of job titles and characteristics of employees in each unit.

**Table 1:** Job title and characteristics of workers

**Workplace**	**Job title**	**Number of people employed**	**Education**
Ethylene substation	Engineer	4	Bachelor
	Technician	4	Associated diploma
	Worker	1	Diploma
Boiler substation	Engineer	2	Bachelor
	Site man	3	Associated diploma
	Operator	4	Diploma
	Operator	16	Bachelor
	Engineer	6	Bachelor

A total number of 40 people are working in a 12-hour irregular shift in the complex, all of whom are men. They are present in almost 10hours of their shift permanently in one place and leave their workplace just to use the restroom, eat, pray, etc. For a closer examination, the employees were divided in two groups of case and control. At each substation, there is more than one occupational group that all people, regardless of the different job titles, are in one room. The number of personnel working at the high voltage substations (Ethylene and Boiler) that are at risk of occupational exposure to magnetic fields was 18 people who were categorized in the case group. In other words, individuals in every substation, despite different job titles, are all in similar working conditions in terms of exposure to magnetic fields.

In the control group, those 22 people who are at the same working conditions and not exposed to occupational exposure to EMFs were categorized. Job titles in the control group include operators and engineers working respectively in the Control Room and Engineering Room. They were only exposed to background level of EMFs. In order to examine possible relationship between exposures to EMFs and sleep quality, the PSQI questionnaire was prepared while the General GHQ was used to detect correlation between exposure to EMFs and general health. The PSQI questionnaire was designed by Buysse et al. (1989) to help distinguish sleep disorders. It contains seven different questions consisting of individual terms of sleep quality, sleep duration, sleep latency, sleep efficiency, useful habits, sleep disturbances, use of sleeping pills and impaired daily function. Each question was given a score of 0 to 3 so that individuals’ total score would range between 0 and 21. According to the questionnaire designers, scores above 5 were considered as poor sleep quality [23].

The short form GHQ questionnaire including 28 questions was used to detect the general health effects of exposure to EMFs By which the despondences were asked about indisposition, discomfort and general health conditions with the main emphasis on their current psychological, physical and social issues. In other words, the questionnaire includes four categories of questions to diagnose somatic symptoms, anxiety and insomnia, social dysfunction and severe depression. The total score for each individual was obtained from the sum of the scores given to each category of the questions in these four fields of area [24]. Questions were scored by the Likert scale. In this research, a score of 23 was considered as the cut-off point of healthy and unhealthy status. According to which, scores higher than 23 was determined as unhealthy state. The above mentioned Standard questionnaires were filled out onsite. Participants were given adequate training before being asked to complete the questionnaires. The filled out questionnaires were then analyzed by MSTATC. The Chi-square, Duncan’s and *t*-test were also used to detect possible relationship between the variables.

All participants provided informed written consent as approved by the ethics committee of Islamic Azad University, North-Tehran Branch.

## Results

According to the obtained results, there was found no measurement point in the complex with the magnetic field amplitude higher than the standard limit of 1200 μT recommended by ACGIH. The highest field strength was 49.90 μT measured at the Boiler Unit at. The results from the Questionnaires PSQI and GHQ are listed in Table 2.

**Table 2:** The results of the questionnaires by the control and case groups

	**Place**	**Job title**	**PSQI**	**GHQ**	**B**_**max **_**(μT)**
**Control group**	Control room	Operator	2.87	9.5	0.14
	Engineering room	Engineers	1.83	5.5	0.05
**Case group**	Ethylene substation	Engineer	14.33	31.5	40.43
		Technician	4.75	16.75	
		Worker	4	7	
	Boiler substation	Engineer	12.5	20	49.90
		Site man	9	23	
		Site operator	5.5	12.25	

Data analysis by the *t*-test showed a significant difference between the case and control groups in terms of sleep quality at confidence level of 1%. A significant difference was also found between the case group and the control group in terms of general health. The comparison results of these two groups in terms of sleep quality and general health are given in Tables 3 and 4.

**Table 3:** Comparison of the control and case groups in terms of sleep quality and general health

	**Group**	**Mean**	**Variance**	**Standard deviation**
Sleep quality	Case	9.25	20.51	4.53
	Control	2.63	1.13	1.06
General health	Case	7.50	11.14	3.34
	Control	20.70	77.17	8.78

**Table 4:** **Results of the overall evaluation of data by *t*-test**

	**Sleep quality**	**General health**
Variance of the difference between the means	1.1663	10.3937
Standard deviation of the differences	1.0799	3.2239
t’ value	−6.1346	−4.0944
Probability of t’	0.0000	0.0004

The results showed that 61% of workers in the case group suffered from poor sleep quality while 95.5% of those in control group had a good sleep quality and just 4.5% of them had poor sleep quality. The analysis results of the GHQ questionnaires showed that 72% of workers in the case group and all participants of the control group had a good condition of general health. The significant difference between the variance of the variables made it possible to compare the average of results obtained from the questionnaires filled out by each group members.

The Duncan Method was used to compare the mean values. Table 5 shows the outputs from the Duncan Method.

**Table 5:** Results of Duncan method

**Job title**	**Mean of quality of sleep**	**Class**	**Job title**	**Mean of general health**	**Class**
Engineers of Ethylene Unit	14.25	a	Engineers of Ethylene Unit	31.50	a
Engineers of Boiler Unit	12.75	ab	Site man of Boiler Unit	23.00	b
Site man of Boiler Unit	9.00	bc	Engineers of Boiler Unit	20.00	bc
Operator of Boiler Unit	5.50	cd	Technician of Ethylene Unit	16.75	c
Technician of Ethylene Unit	4.75	cd	Operator of Boiler Unit	12.25	d
Worker of Ethylene Unit	4.00	d	Operator of Control Room	9.50	de
Operator of Control Room	3.00	d	Worker of Ethylene Unit	7.00	e
Engineers of Engineering Room	2.25	d	Engineers of Engineering Room	5.50	e

The PSQI analysis results by Duncan’s Method revealed only three types of occupational groups including “workers” of the Ethylene Unit, “operators” of the Control Room and “engineers” of the “Engineering Room” enjoyed were categorized into the lowest class of “d” (Table 5) and enjoyed good quality sleep. Compared to other occupational groups, engineers at the Ethylene Unit had the worst sleep conditions and classified in the highest Dunkan class of “*a*”. The sleep quality of those working in charge of engineers, site man and operator at Boiler Unit was poor, as well.

Accordingly, the occupational group of engineers at Boiler Substation was classified between the classes “*a*” and “*b*” symbolized as *ab* class. However, the occupational groups “site man” and “operators” of the unit were categorized in lower classes at a confidence level of 1%.

Although, the mean value calculated for sleep quality of technicians at Ethylene Unit was lower than the index value however, they were given a score similar to those working at Boiler Unit so as all were classified at the same class. Both numbers, with a very little difference, are close to the index number of 5, it can be concluded that these people are prone to crisis.

The analysis results of GHQ questionnaires showed that engineers working at Ethylene Unit were categorized in the highest class while those engineers working at Engineering Room were classified in the lowest class.

Only scores obtained for engineers at Ethylene Unit and site men of the Boiler Unit were higher than the index number of 23. This indicates personnel in these groups do not enjoy good health conditions. However, those in other occupational groups either in case group or particularly in the control group were given scores lower than the index number that indicates they are in a good conditions of health. The analysis results of the Chi-square Test for the PSQI questionnaires showed that the score of “F” was significant at the confidence level of 1% for operators at the Boiler Unit and Control Room as well as engineers at Engineering Room and technicians of the Ethylene Unit. In each occupational group, data distribution is at an acceptable level and sleep quality of the individuals does not differ much.

As the results suggest, the “F” score was not significant at confidence level of 1 for Engineers at Ethylene Unit operators and site men at Boiler Unit. Consequently, the results obtained for sleep quality of every individual differ and the data distribution is high. The “F” score of the GHQ questioners was found insignificant for all job titles at confidence level of 1%. This revealed different general health condition of the employees where as data distribution is so high.

## Discussion

As mentioned earlier, there was found no measurement point at which magnetic field amplitude value exceeds limits recommended by ACGIH. It should be noted that the maximum EMF measured in this research was 49.90 μT. In a similar study by Sharifi Fard et al. (2011), there was also found no evidence of impermissible EMF at any point measured; the highest recorded values were 0.69 μT and 9.15 μT recorded among the Control Rooms and near the switchgear, respectively [25]. Their findings showed no significant difference between the case and control groups in terms of psychological disorders [19].

Another investigation on the effect of extremely low frequency electromagnetic fields exposure on sleep quality in high voltage substations in city of Kerman and the suburbs shows that although there was a higher percentage of poor sleep quality on the case group than the control group, no statistically significant difference was perceived [26].

The results of the present study also show that compared with those in control group, the individuals who have been in occupational exposure to magnetic fields (the case group), may have poorer sleep quality and worse health status. However, no significant correlation was observed between exposure level and sleep quality and general health condition of the personnel (p < 0.01). Yousefi and Nassiri in 2006 reported that symptoms of mental and behavioral disorders among people exposed to EMFs were significantly higher than those in control group. However, they could find no significant correlation between the above-mentioned symptoms and field strength and duration of exposure [18]. A case–control study by Wijngaarden *et al*. on exposure to EMFs showed that suicide rate was higher among those exposed to EMFs which could be due to the prevalence of depression among this group [27]. In a study by Zahiroddin *et al.* it was demonstrated that 17% of the control group and 32.7% of the case group were suspected of failing health (p < 0.01) and it was indicated that incidence of mental health disorders have no relationship with variables such as marital status, age, work experience and shift work [28]. Gamberala *et al*. found symptoms of depression, paranoid, obsessive-compulsive disorder, sensitivity in individual and social behaviors, anxiety and aggression among workers exposed to EMFs [29]. Another study by Pearce *et al*. showed psychological side effects such as suicide, depression and uncontrollable emotional state in people living near power transmission lines and high voltage substations [30]. The findings of the present study on insignificant correlation of exposure level and sleep disorder and general health may be due to the small sample size of the study groups. Accordingly, further studies on larger populations are required. As mentioned earlier, the case group in this study included personnel of Ethylene and Boiler Units. In each unit, there were three job titles with similar working conditions and almost the same exposure level to magnetic fields. In spite of the similarity in working conditions and exposure level at each unit, the sleep quality and health status of each occupational group differ from each other (p < 0.01). This is probably due to differences in physical and mental characteristics of the personnel. Employees with higher education degrees scored higher for sleep quality and general health status, showing that they had more undesirable quality of sleep and health condition than other staff. Zamanian et al. also revealed the psychological health level of people exposed to ELF-EMFs was lower than that of control group. They could also find a significant correlation between deep depression and education level [31]. Despite numerous studies on the effects of EMFs on human, scientists have still failed to reach a consensus on the real side effects. Given the complexities of biological, psychological and social factors affecting human health, detailed examination on the effects of EMFs requires further researches at a larger scale.

## Conclusion

The present study was done to investigate possible correlation between ELF EMFs and the general health and sleep quality of employees working at two power substations in a petrochemical company in southern Iran. As the findings suggest, in spite of a significant difference between the case and control groups in terms of sleep quality and general health, no significant relationship was found between the exposure level and sleep quality and general health. Although the amplitude of the magnetic fields was evaluated lower than the standard limits at all measurement points, however, more comprehensive studies should be done to detect possible side effects may cause by exposure to even below-standard EMFs. It is recommended to reduce exposure to the EMFs as much as possible through strategies such as increasing distance from EMF sources by remote control devices, training exposed workers and planning to diminish workplace stress.

## Competing interests

The authors hereby declare that they have no competition of interests to be told.

## Authors’ contributions

MRM: conception and design, analysis and interpretation, administrative, technical, or material support, supervision. MH: conception and design, data collection,analysis and interpretation, statistical expertise, writing the manuscript, critical revision of the manuscript. LFL: conception and design, administrative, technical, or material support.HAA: writing the manuscript. HKH: administrative, technical, or material support. AH: data collection, technical, or material support. All authors read and approved the final manuscript.
